# Effects of obesogenic diet and estradiol on dorsal raphe gene expression in old female macaques

**DOI:** 10.1371/journal.pone.0178788

**Published:** 2017-06-19

**Authors:** Cynthia L. Bethea, Kevin Mueller, Arubala P. Reddy, Steven G. Kohama, Henryk F. Urbanski

**Affiliations:** 1 Division of Reproductive and Developmental Science, Oregon National Primate Research Center, Beaverton, OR, United States of America; 2 Division of Neuroscience, Oregon National Primate Research Center, Beaverton, OR, United States of America; 3 Department of Obstetrics and Gynecology, Oregon Health and Science University, Portland, OR, United States of America; 4 Department of Internal Medicine, Texas Technical University Health Sciences Center School of Medicine, Lubbock, TX, United States of America; 5 Department of Behavioral Neuroscience, Oregon Health and Science University, Portland, OR, United States of America; Pennsylvania State University College of Medicine, UNITED STATES

## Abstract

The beneficial effects of bioidentical ovarian steroid hormone therapy (HT) during the perimenopause are gaining recognition. However, the positive effects of estrogen (E) plus or minus progesterone (P) administration to ovariectomized (Ovx) lab animals were recognized in multiple systems for years before clinical trials could adequately duplicate the results. Moreover, very large numbers of women are often needed to find statistically significant results in clinical trials of HT; and there are still opposing results being published, especially in neural and cardiovascular systems. One of the obvious differences between human and animal studies is diet. Laboratory animals are fed a diet that is low in fat and refined sugar, but high in micronutrients. In the US, a large portion of the population eats what is known as a “western style diet” or WSD that provides calories from 36% fat, 44% carbohydrates (includes 18.5% sugars) and 18% protein. Unfortunately, obesity and diabetes have reached epidemic proportions and the percentage of obese women in clinical trials may be overlooked. We questioned whether WSD and obesity could decrease the positive neural effects of estradiol (E) in the serotonin system of old macaques that were surgically menopausal. Old ovo-hysterectomized female monkeys were fed WSD for 2.5 years, and treated with placebo, Immediate E (ImE) or Delayed E (DE). Compared to old Ovx macaques on primate chow and treated with placebo or E, the WSD-fed monkeys exhibited greater individual variance and blunted responses to E-treatment in the expression of genes related to serotonin neurotransmission, CRH components in the midbrain, synapse assembly, DNA repair, protein folding, ubiquitylation, transport and neurodegeneration. For many of the genes examined, transcript abundance was lower in WSD-fed than chow-fed monkeys. In summary, an obesogenic diet for 2.5 years in old surgically menopausal macaques blunted or increased variability in E-induced gene expression in the dorsal raphe. These results suggest that with regard to function and viability in the dorsal raphe, HT may not be as beneficial for obese women as normal weight women.

## Introduction

Today women face two major health issues, menopause and obesity, both of which seriously impact age-related diseases and quality of life. Proper hormone therapy (HT) could ameliorate the onset and severity of deterioration, but it has been mired in controversy since the Women’s Health Initiative (WHI). Subsequently, the use of human estradiol (E), as well as administration during the perimenopause, have yielded positive effects on mood [1,2], cognition [3], carotid intima-media thickness (CIMT; [4]), metabolism and body composition [5], lung function [6] and immune function [7] (including multiple sclerosis [8]), compared to no treatment or delayed treatment. In contrast, the ELITE trial reported no difference in cognitive function in women taking oral E within 4 years of menopause or after 10 years of menopause [9]. Nonetheless, bio-identical HT administered during a ‘window of opportunity’ shows promise in some systems for subgroups of women who are not at risk for hormone-sensitive malignant conditions, counter-indications (American Association of Clinical Endocrinologists) or other adverse life events [2].

In the US, a large portion of the population eats what is known as a Western Style Diet (WSD) that is high in fat and refined sugar. Specifically, 2/3 of the US population is overweight or obese [10], while 114 million people exhibit symptoms of pre- or frank diabetes (American Diabetes Association). As obese women enter menopause, an obvious speculation is that their risk for disease could increase over the presence of either risk factor alone [11]. Obesity and WSD cause an increase in systemic cytokine production that in turn, negatively impacts many functions [12–18]. Notably, obesity and its adjunct cytokines have been linked to depression and neurodegeneration [19–28].

The serotonin system plays a supportive role in many aspects of neural function and E, with or without P, increased genes or proteins that should improve serotonin neurotransmission and serotonin neuron viability in macaques [29–32]. These referenced studies were conducted in a cost effective model of surgical menopause in adult female macaques (7–13 years) administered HT for 1 month within 5 to 8 months after ovariectomy (Ovx). Recently, we found that 4 years of E replacement (ERT) shortly after Ovx in old macaques (>20 years) increased gene expression related to serotonin neurotransmission and viability in the same manner as in younger adults treated for 1 month [33]. However, the adult and old macaques were maintained on monkey chow, fresh fruit and vegetables. Monkey chow is low in fat and sugar, but high in micronutrients; and it bears no resemblance to WSD consumed by a majority of Americans. In addition, HT was initiated well within the ‘window of opportunity’ allowing that 1 monkey year equals 3 human years.

In this study, we used qRT-PCR to examine E-regulated gene expression in the serotonergic dorsal raphe of old (>20 yrs) rhesus macaques maintained on WSD. The animals were ovo-hysterectomized (OvH; spayed; surgically menopausal) and administered (1) placebo, (2) immediate estradiol (ImE) or (3) delayed estradiol (DE) at 2 years past OvH.

Focus was placed on genes related to serotonin function, cellular resilience or viability, and neurodegenerative diseases. The serotonin related genes were selected from earlier experiments that demonstrated their regulation by ovarian steroids at gene and protein levels [34]. Dendritic spine extrusion and synapse assembly play pivotal roles in neuronal plasticity. Therefore, genes were selected that related to dendritic spines and synapse assembly that had shown regulation in adult and old chow-fed monkeys [33,35,36]. We also showed that serotonin neurons in placebo-treated Ovx animals had more DNA fragmentation (TUNEL positive) than animals with hormone therapy for 1 month [31]. DNA fragmentation and other neurodegenerative mechanisms are controlled by DNA repair enzymes, chaperones (protein folding), ubiquinases, transport motors and mutations within specific genes. Therefore, we selected genes related to these categories and that previously showed regulation by E in adult and old chow-fed females [32,33]. Finally, 3 E-regulated genes with known roles in neurodegenerative disease (NDD) were examined as previously described [32,33].

## Materials and methods

This experiment was approved by the IACUC of the Oregon National Primate Research Center and conducted in accordance with the 2011 Eighth Edition of the National Institute of Health *Guide for the Care and Use of Laboratory Animals*.

### Animals

The old rhesus macaques (>20 years) used in this study were largely experimentally naïve retired breeders of Indian pedigree. The animals were provided with unlimited access to WSD pellets (TAD; Lab Diet, Inc., St. Louis, MO) plus fresh fruit and vegetables. The animals maintained on monkey chow were previously described in detail [33]. Briefly, they were also old rhesus females provided with biscuits from Lab Diet, Inc. to maintain a healthy body weight plus fresh fruit and vegetables. All animals had unlimited access to drinking water via lick-it spouts. Technicians and clinical veterinarians in the Department of Comparative Medicine monitored the animals daily. Regular monkey chow provides calories with 13% fat, 69% complex carbohydrates (includes 6% sugars) and 18% protein. WSD provided calories with 36% fat, 44% carbohydrates (includes 18.5% sugars) and 18% protein.

The WSD animals were socially housed in large indoor pens with feeders, foraging mats, toys, shelves, and a tunnel for separation of individuals as needed for administration of medicines, obtaining fasting glucose and insulin measurements, administration of ketamine prior to transport, or for obtaining blood samples. Three or four animals were housed in each pen. Additional enrichment included watching video programs and interactions with the Behavioral Science Unit staff and animal care technicians.

### Diet, ovo-hysterectomy and timing of E treatment

The old female macaques were started on WSD approximately 6 weeks prior to ovo-hysterectomies (OvH; spay). A surgical veterinarian and surgical staff of ONPRC performed OvH via trans-abdominal surgery. OvH was performed because the old female macaques were to receive E for 30 months (2.5 years), and endometrial hyperplasia would be expected. Since older macaques are also more susceptible to cancer, OvH was used to decrease risk during the treatment period.

The old WSD-fed macaques received [1] placebo for 30 months, or [2] E immediately after hysterectomy for 30 months (ImE), or [3] placebo for 24 months and Delayed E for an additional 6 months (2.0 years interval; DE for 6 months). The protocol was started with **8** animals in each treatment group. The animals were organized into cohorts of 8 containing animals with different treatments and different ranks. The treatment protocols were staggered 1 month by cohort to obtain all measurements on all animals in a technically feasible fashion. In the first 6 months of the study, 4 animals were deemed unsuitable. They were replaced and the treatment protocols were further staggered for the 4 late animals. Afterwards, attrition occurred due to age related diseases. The old chow-fed macaques used for comparison to WSD-fed monkeys received [1] placebo (n = 4) or [2] E (n = 3) shortly after ovariectomy (Ovx), which continued for 48 months [33].

E was administered via Silastic capsules implanted subcutaneously in the periscapular region. One Silastic capsule (3.5 to 4.5-cm depending on metabolism; inner diameter, 0.132 in., outer diameter, 0.183 in.; Dow Corning, Mid-land, MI) was packed with crystalline estradiol (1,3,5**_**10-estratrien-3,17—diol, Steraloids, Wilton, NH). The implants were intended to achieve between 70–100 pg/ml in serum.

Serum E was measured every 2 months in the old WSD-fed macaques starting shortly after hysterectomy. Monkey metabolism can be highly variable. At any time during the duration of the protocol, if serum E concentrations were higher than 120 pg/ml in an individual measurement, the capsule was replaced with a smaller capsule, which was used for that individual henceforth. When serum E levels declined below 50 pg/ml the implants were replaced. There is a surge of E immediately after implantation that gradually declines and stabilizes, so monitoring every 2 months enabled us to maintain the goal on average. The general characteristics and average serum E concentrations of the monkeys are shown in **Table 1.**

**Table 1:** Characteristics of the old macaques used for raphe gene expression at the end of the study.

Groups	Surgery	Diet	Age (yrs)	Weight (kg)	Interval to ERT	Length of Treatment	Treatment (n)
							Average Serum E (pg/ml)
Short Interval to ERT					2 mo	48 mo	Placebo (n = 4)
							<30
	Ovx	Chow	26.6±1.21	7.47±0.25			
							ERT (n = 3)
							94.3±20.5**
	No Interval to ERT	OvH	WSD					0 days	30 mo	Placebo (n = 5) 6.60 ± 0.16
			21.29 ± 0.32	8.99 ± 0.31			
					0 days	30 mo	ImE (n = 6) 82.05 ± 0.93*
Long Interval to ERT	OvH	WSD			2.0 yrs	6 mo	DE (n = 6)
							90.78 ± 1.29*
							

*ANOVA p < 0.001

** t-test p<0.001

### Other protocols

During the treatment period, other measurements were obtained at either 6-month or yearly intervals from the WSD-fed animals as follows: body weight, Glucose Tolerance Test (IVGTT), fasting glucose and insulin, Dexascans, hyperinsulinemic-euglycemic clamps, MRI with ^1^H-spectropscopy on liver and muscle, MRI of brain, MRI of brain with vessel contrast dye, sleep-wake activity with Actiwatch collars, focal observations of numerous behaviors based upon a common ethogram, Human Intruder and Novel Object Tests, Spatial Maze test, retinal fundus autofluorescence of lipofuscin accumulation, ultrasound of carotid arteries and heart, core body temperature, blood samples for basal immune function and following vaccination. Blood samples were also obtained at 6-month intervals in the morning and evening for cortisol, DHEAS, leptin, and melatonin. Samples for determination of 9 different cytokines were obtained in the beginning and at the end of the project period. These data are in preparation for multiple publications.

### Steroid hormone assays

The ONPRC Endocrine Technology and Support Core performed E assays using a Roche Diagnostics Cobas e411 automatic clinical platform assay instrument. The sensitivity limit of the E assay was 5 pg/ml. The intra-assay and inter-assay coefficients of variation were all less than 10%. Prior to these analyses, measurements of E on this platform were compared to traditional RIA’s as previously reported [37].

### Euthanasia

The monkeys were euthanized at the end of the treatment periods according to procedures recommended by the 2013 Edition of the American Veterinary Medical Association *Guidelines for the Euthanasia of Animals*. Each animal was sedated with ketamine, administered pentobarbital (30 mg/kg, i.v.), and exsanguinated by severance of the descending aorta.

### Tissue preparation

The brain was removed from the cranium and dissected into blocks. The midbrain block was removed. Externally, the block extended from the anterior to the caudal end of the pons. Internally, the block extended from the caudal linear nucleus to the locus coeruleus. The midbrain raphe block was then frozen in liquid nitrogen and stored at -80^°^C.

### qRT-PCR assays

RNA extraction and qRT-PCR assays were executed in a manner similar to a previous experiment with chow-fed animals. Upon removal from storage, the raphe block was microdissected to a small piece containing the dorsal raphe. The dorsal raphe block was immediately dropped in TriReagent and further cleaned with a Qiagen RNAeasy column (Velencia, CA). Total RNA (260/280) from the individual animals and from a standard pool of rhesus tissues was stored at -20^°^C at an average concentration of 6.8 ng/μl. Reverse transcription and complementary DNA (cDNA) synthesis was performed using RNA-to-cDNA kit from ABI (ThermoFisher, Foster City, CA) containing RT Enzyme Mix and RT Buffer Mix according to the protocol of the manufacturer. The quantity of cDNA recovered was determined with fluorescent spectrometry (Qubit, ThermoFisher, Waltham, MA). The cDNA yield averaged 0.263 ng/μl. Pre-amplification of all samples and the standard pool was conducted with Taqman PreAmp Master Mix and an ABI custom pooled primer mix for 14 cycles. The product was diluted five-fold for use in the Taqman Expression Array. The concentration of cDNA in the dilution averaged 4.48 ng/μl.

Monkey specific primers from the ABI Rhesus Monkey Library (Applied Biosystems, Foster City, CA) were preloaded in triplicate on custom 384-well Taqman Expression cards for qPCR. The primers utilize a 5’ fluorescent reporter, FAM (Fluorescein amidite; Molecular Probes, Eugene, OR) and a 3’ quencher, TAMRA (tetramethylrhodamine), which improves sensitivity. The expression of 31 genes was examined. The exact primer sequences are proprietary, but the gene names, symbols, AB assay IDs and NCBI gene reference information are shown in **Table 2**.

**Table 2:** Available information about the ABI flourescent primers used in this study.

Gene Name	Gene Symbol	Assay ID	NCBI Gene Reference
*Serotonin regulation*			
Fifth Ewing variant (PET1 in rodent)	*FEV*	Rh02872593	XM_001095962.2
Tryptophan hydroxylase 2	*TPH2*	Rh02788839	NM_001039946.1
Serotonin reuptake transporter	*SLC6A4*	Rh02787892	NM_001032823.1
Serotonin receptor 1A	*5HT1A*	Rh02902683	NM_001198700.1
*CRH System*			
Corticotropin releasing hormone receptor type 1	*CRHR1*	Rh02787591	NM_001032803.1
Corticotropin releasing hormone receptor type 2	*CRHR2*	Rh01120857	XM_001085987.2
Urocortin 1 (stresscopin)	*UCN1*	Rh03986716	NM_001265661.1
*Synapse assembly*			
Neuroligin 3	*NLGN3*	Rh03986723	XM_001111843.1
Neurotrophic tyrosine kinase, receptor, type 2, *trk*-B	*NTRK*	Rh02831788	NM_001261297.1
*DNA Repair*			
Nibrin, part of double strand break repair complex	*NBN (NBS1)*	Rh01039845	XM_001085033.1
Nth endonuclease III-like 1, DNA N-glycosylase	*NTHL1*	Rh00959765	XM_001082772.2
Ligase IV, DNA double strand break repair	*LIG4*	Rh04269856	XM_001084107
RAD23 homolog A, involved in nucleotide excision repair(NER)	*RAD23A*	Rh00908422	XM_001110103.2
Apurinic/apyrimidinic endonuclease 1	*APEX1*	Rh02793202	XM_001090240.2
Proliferating cell nuclear antigen, BER gap filling	*PCNA*	Rh02806147	XM_001115746.2
*Chaperones*			
Heat shock protein 90kD	*HSP90B1*	Rh02790147	NM_001195524.1
Heat shock protein 60kD protein1	*HSPD1*	Hs01036753	NM_002156.4
Heat shock protein 27kD protein 1	*HSPB1*	Rh02980144	XM_001109274.2
*Ubiquinases*			
Ubiquitin-like modifier activating enzyme 1	*UBE1*	Hs01566989	NM_024818.3
Ubiquitin-conjugating enzyme E2D 3	*UBE2D3*	Hs01518517	NM_181892.2
Ubiquitin protein ligase E3A	*UBE3A*	Rh00963674	XM_002804686.1
*Transport*			
Kinesin family member 5B	*KIF5B*	Rh01037194	XM_002805607.1
Dynein, light chain, LC8-type 1	*DYNCL1*	Hs00867659	NM_001037495.1
Microtubule-associated protein tau	*MAPT*	Rh04269822	XM_001115803.2
*Disease specific*			
α-secretase cleavage of APP precludes amyloid-β formation	*ADAM10*	Rh01109565	XM_001097016.2
α-synuclein, presynaptic signaling, membrane trafficking	*SNCA*	Rh1103386	XM_001095402.2
Presenilin (γ-secretase component) produces amyloid-β 40–42	*PSEN1*	Rh02826228	XM_001088635.2

Four concentrations of the standard pool (100μl) and 100μl of cDNA (~4.5 ng/μl) from the preamp dilution were mixed with 100μl of ABI Universal Master Mix and loaded into each slot. The sample was distributed across 48 wells yielding 0.933 ng/well of cDNA. There was a log linear increase in fluorescence detected as the concentration of amplified double-stranded product cDNA increased during the reaction. The fluorescence was detected as cycle threshold (Ct) with an ABI 7900 thermal cycler (Applied Biosystems Inc.) during 40 cycles. For each transcript, the slope of the standard curve (y) was obtained and used to calculate the relative ng of each transcript (10^y) in the cDNA from each raphe. The sample ng were normalized by the geometric mean of 18s cDNA X GAPDH cDNA. The only difference between the current experiment with WSD-fed monkeys and the previous experiment with chow-fed monkeys included the use of newer ABI Master Mixes instead of individual reagents.

We chose to examine a technically reasonable number of genes in triplicate that would be representative of complex functions. The genes selected were regulated in laser captured serotonin neurons from adult Ovx females ± HT for 1 month [32,35,36,38,39] and in a small block of midbrain containing the dorsal raphe from old Chow-fed Ovx macaques ± HT for 4 years [33]. The number of wells available on the Taqman card, which also contained internal controls, determined the number of genes examined.

### Genes examined and reported

Serotonin regulation- *FEV* (Fifth Ewing variant, determines serotonin phenotype), *TPH2* (tryptophan hydroxylase, rate limiting enzyme in serotonin synthesis, *SERT* (serotonin reuptake transporter), *5HT1A* (serotonin 1A receptor), *IDO* (indoleamine 2, 3-dioxygenase).

CRH system- *CRHR1* (corticotropin releasing hormone receptor type 1), *CRHR2* (corticotropin releasing hormone receptor type 2) and *UNC1* (urocortin 1, stresscopin).

Synapse assembly- *NLGN3* (neuroligin 3) and *NTRK2* (TrkB, neurotrophic tyrosine kinase),

DNA repair- related–*NBN* (nibrin, double strand break repair complex), *NTHL1* (endonuclease family III), *LIG4* (DNA double-strand break repair, nonhomologous end joining), RAD23A (double strand break repair; DNA damage recognition), *APEX1* (multifunctional DNA repair), and *PCNA* (single base repair).

Chaperones–heat shock proteins *HSP90B1 (HSP90)*, *HSPD1 (HSP60)*, *HSPB1 (HSP27)*.

Ubiquinases–*UBEA5* (UBE1*;* type E1 activating), *UBE2D3* (type E2 conjugating), *UBE3A* (type E3 ligase).

Transport motors—*KIF5B* (kinesin), *DYNCL1* (dynein), *MAPT* (tau).

Disease specific genes—*ADAM10* (α-secretase), *PSEN1* (component of γ-secretase), SCNA (α- synuclein).

Glyceraldehyde 3-phosphate dehydrogenase (*GAPDH*), 18S, and β-actin were measured for reference and normalization.

### Statistical analysis

GAPDH and 18s were not altered by treatment, but β-actin was altered by E treatment in the chow-fed animals. Based upon a compelling analysis, we used the geometric mean of 18s and GAPDH for normalization of the expression results [40]. The geometric mean equals the square root of the product of 18s X GAPDH cDNAs. In order to compare the results to chow-fed macaques treated in a similar fashion, the data from a previously published study [33] were also normalized by the geometric mean of 18S X GAPDH cDNAs. The earlier data from the chow-fed monkeys was published with normalization by GAPDH cDNA only, so this paper contains different, albeit similar, results. The mean of the normalized result of the triplicates was considered the individual animal’s result. The average of the results of individual animals in a group was used in statistical analyses. Thus, the standard error of the mean represents within group variance between animals. The monkeys maintained on WSD were euthanized approximately 7 years after the chow-fed monkeys were euthanized. However, the Taqman Expression Arrays with identical ABI primers were conducted on the chow-fed monkeys in June 2014 and on WSD-fed monkeys in December 2016. The brain tissue blocks were kept frozen at -80^°^C for the interim from necropsy to TriReagent extraction.

The chow-fed animals were treated with placebo, E and E+P for 4 years. There was no DE group in the chow-fed experiment, and there was no E+P group in the WSD-fed experiment. Therefore, the data from only chow-fed placebo and E-treated groups were used for comparison to the WSD results. In order to statistically determine the interaction of treatment and diet, a 2-way ANOVA with the Bonferroni post-hoc test were applied. However, the 2-way ANOVA required an equal number of groups, so it necessitated exclusion of the DE group. Therefore, the average relative expressions of all of the WSD-fed groups were analyzed with 1-way ANOVA followed by Newman-Keuls posthoc pairwise comparisons (3 groups); On 2 occasions the chow-groups were not different with Bonferroni, but were different by 1-way ANOVA in the previous report [33]. Therefore, we applied and additionally report the results of a t-test on the data from the chow-fed groups The analysis was designed to ask- did E treatment cause a statistically significant difference in mRNA expression compared to placebo in WSD -fed or chow-fed animals?

Variance between animals is not unusual for this type of preparation, but the variance in the WSD animals exceeded that of previous studies with chow-fed animals. Each of the WSD-fed groups started with 8 animals. However, there was higher attrition in the WSD-fed placebo group that reduced the group to n = 4 prior to qRT-PCR. The ImE group and the DE group each contained 6 animals. Attrition also occurred in the experiment with old chow-fed monkeys so that there were 4 animals in the placebo group and 3 animals in the E-treated group for gene expression analysis. Prism 5.0 from Graph Pad (San Diego, CA) was used for statistical tests. Comparisons were considered significantly different when the chance of making a type 1 error was less than 5% (p<0.05). We recognize that with a small ‘n’, the chances of making a type 1 error (stating that there is no difference between groups when there is a difference in the larger population) are increased. Therefore, trends are noted in light of the possibility of type 1 error. The results from the chow-fed animals were previously subjected to a multiple comparison False Discovery Rate (FDR) procedure using the Benjamimi-Hochberg test for False Discovery Rate ([33]. However, in the WSD animals only 3 genes showed a significant regulation by ImE or DE so FDR was not applicable. Please note that Prism 5 provides an exact p-value when ‘p’ is higher than 0.0001, but p-values less than 0.0001 are designated as such, and thus, are not exact. In addition, p-values for posthoc tests are expressed as less than 0.05, 0.01 or 0.001 rather than exact values.

## Results

### Animals

The clinical veterinarians closely monitored these geriatric animals and any sign of illness or discomfort was treated aggressively. The most common pathology was GI tumors that required removal and resection of the small or large intestine. This surgery extended the life span of the individual, but not to the end of the protocol in several cases. Other causes of euthanasia included a stroke, paralysis of both legs from unknown etiology, cancer, bladder malfunction or trauma from a fight. Non-lethal illnesses that were treated successfully included dermatopathies, joint disease, diarrhea and constipation. The diseases requiring euthanasia prior to the end of the protocol were considered ‘stressors,’ and deemed exclusion criteria for raphe gene analysis. At the end of the protocol, 4 placebo-treated, 6 E-treated and 6 DE treated animals were included in the raphe gene analysis. Unfortunately, one of the placebo samples had very poor yield of mRNA and could not be used on the Taqman Gene Expression Array.

All animals had Dexascans prior to all protocols and every 6 months thereafter. The percent of total mass that was fat increased from 17.09±0.40% at baseline (all animals) to 39.73±2.72%, 39.14±2.97% and 40.91±3.41% in placebo, ImE and DE groups, respectively. There was no difference in %fat mass between the groups at the end of 30 months. However, the ImE group progressed to obesity at a slower rate than placebo or DE groups (in preparation for publication).

### General

Gene expression was determined with qRT-PCR in a micro-dissected block of midbrain containing the serotoninergic dorsal raphe nucleus from old OvH macaques treated with placebo (n = 3), or ImE (n = 6) or DE (n = 6) over 2.5 years (7.5 human years). The data was collated to obtain the mean ratio of gene/ [SQRT(18s X GAPDH)] for each animal. The mean of multiple animals from each treatment group were statistically compared. A total of 31 cDNAs were examined, 28 cDNAs were detectable and the ratios of 27-cDNAs/geometric mean are shown in the figures. In addition, the normalized ratio of gene expression in the WSD-fed animals was compared to chow-fed animals. Although there were several exceptions, overall transcript abundance was frequently reduced in WSD-fed animals compared to chow-fed animals. The concentration of cDNA reflects the starting concentration of mRNA and it is the regulation of mRNA that is of interest. Therefore, results will be discussed as mRNA henceforth.

### Steroid hormone concentrations achieved by implants

**Fig 1:**
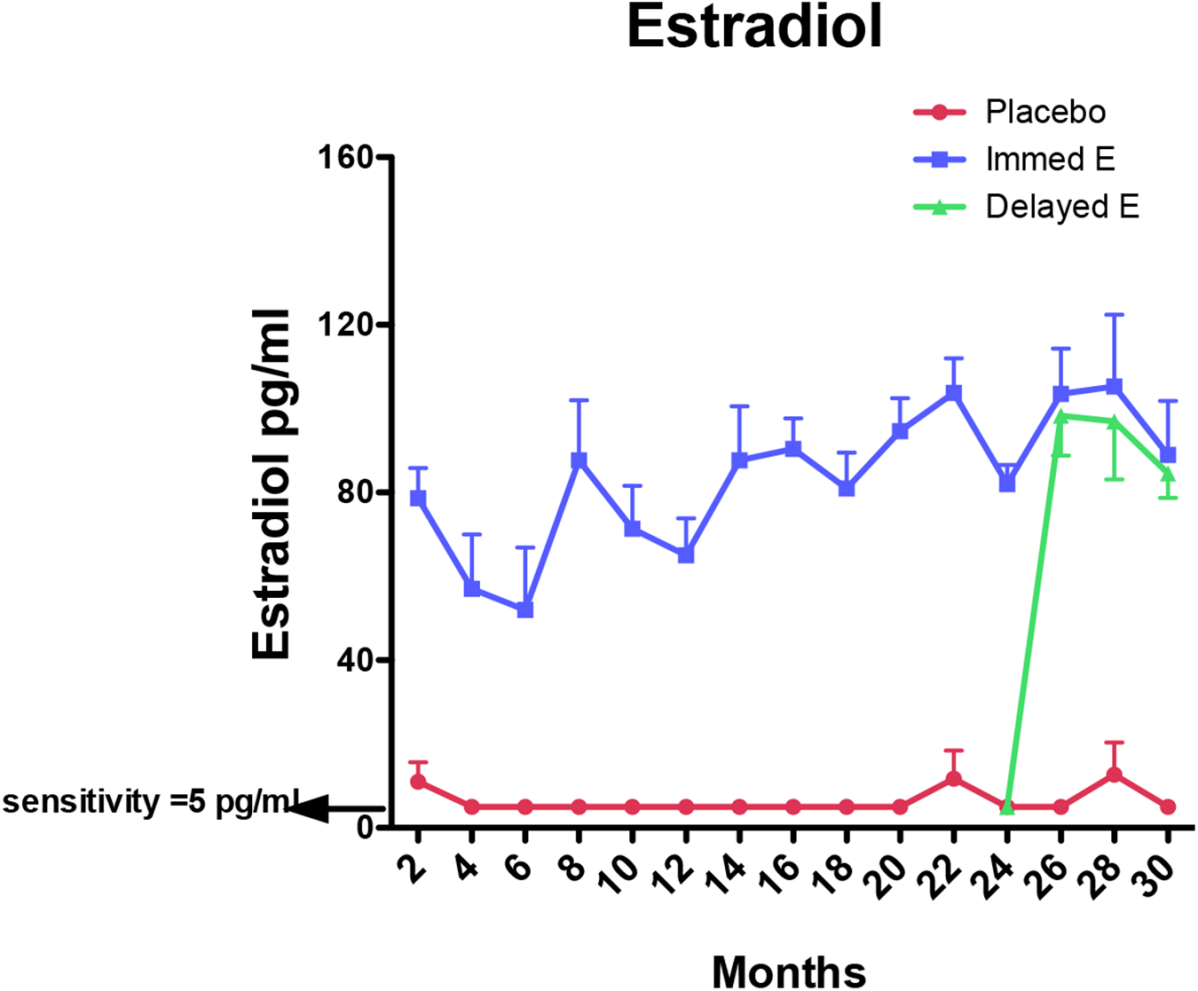
Illustration of serum estradiol (E) concentrations (pg/ml) in the 3 groups of old OvH WSD-fed macaques treated with placebo, ImE or DE over the course of 30 months. Placebo and ImE were initiated at the end of the OvH surgery. DE was initiated after the 24-month measurements and continued for 6 months. Serum E concentrations were significantly higher in the ImE group than in the placebo and DE groups from months 1–24 (2-way ANOVA p < 0.0001). Serum E concentrations were similar in the ImE and DE group from months 24–30, and both ImE and DE groups were significantly higher than placebo during the same time period (p<0.0001).

**Fig 1** illustrates the average bi-monthly serum E concentrations in the old WSD-fed macaques. E-filled Silastic implants significantly elevated serum E concentrations compared to placebo controls (2-way ANOVA p < 0.0001). In the WSD-fed groups, serum E concentrations in the placebo group averaged 6.60±0.16 pg/ml. In the ImE and DE groups, serum E concentrations averaged 82.05±0.93 pg/ml and 90.78±1.29 pg/ml, respectively, during the E-treatment periods (**Table 1**).

In the chow-fed groups, serum E concentrations in the placebo group averaged <30 pg/ml and often fell below the limit of assay sensitivity (at the time of the experiment a less sensitive assay was in use). In the chow-fed E-treated animals, the average serum E concentration during the final two years of the study was 118.8 ± 15.9 pg/ml, and at the time of tissue collection, E equaled 94.3 ± 20.5 pg/ml.

Although the serum E concentrations were slightly higher than those observed in the very early follicular phase or achieved by vaginal rings in women (60–70 pg/ml), the treatments were identical to that used in earlier studies with young adult females. The achieved concentrations have produced consistent experiment-to-experiment increases in TPH2 mRNA (internal control) in chow-fed animals. These concentrations of E fall far below the concentrations observed during the ovulatory surge (400–1000 pg/ml) in macaques.

### Serotonin regulation

**Fig 2:**
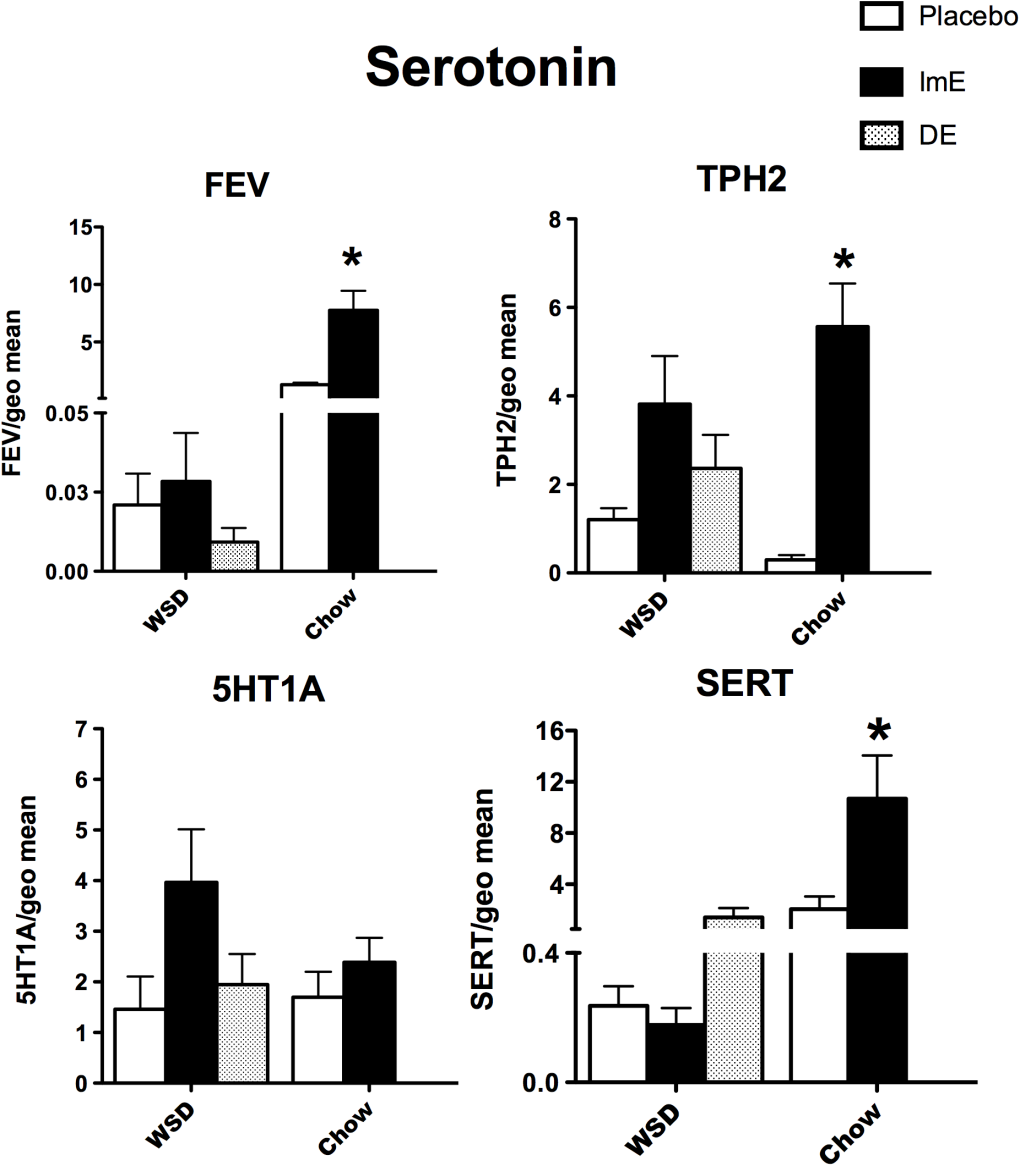
Histograms illustrating the expression of 4 genes involved in serotonin neurotransmission. FEV, TPH2 and SERT were significantly increased by E treatment in the chow-fed monkeys. WSD prevented FEV, TPH2 and SERT from responding to E (1-way ANOVA). There was a significant interaction between diet and treatment in FEV and SERT expression. Transcript abundance of FEV and SERT was markedly lower in WSD-fed monkeys, whereas TPH2 and 5HT1A mRNAs had similar abundance in both diets. * Bonferroni post hoc p<0.001.

The relative mRNA expression of 4 genes known to have pivotal roles in the regulation of serotonin neurotransmission is illustrated in **Fig 2**. FEV and SERT mRNAs showed an effect of diet (p<0.0001; p = 0.0007), an effect of E treatment (p = 0.0002; p = 0.0079) and an interaction between diet and E treatment (p = 0.0002, p = 0.0074) with 2-way ANOVA. TPH2 showed an effect of E treatment (p = 0.0014), but no effect of diet and no interaction. FEV, TPH2 and SERT mRNAs were higher with E treatment in the chow-fed monkeys (Bonferroni, p<0.01). 5HT1A expression was not different between the groups with 2-way ANOVA analysis. In WSD-fed monkeys analyzed with 1-way ANOVA, there was no difference between the groups in FEV, TPH2, 5HT1A or SERT expression (p = 0.46; p = 0.23; p = 0.14; p = 0.17, respectively). FEV and SERT mRNA abundance was an order of magnitude lower in the WSD-fed compared to the chow-fed monkeys. TPH2 and 5HT1A mRNAs were of similar abundance in the 2 diets.

### CRH system in the midbrain

**Fig 3:**
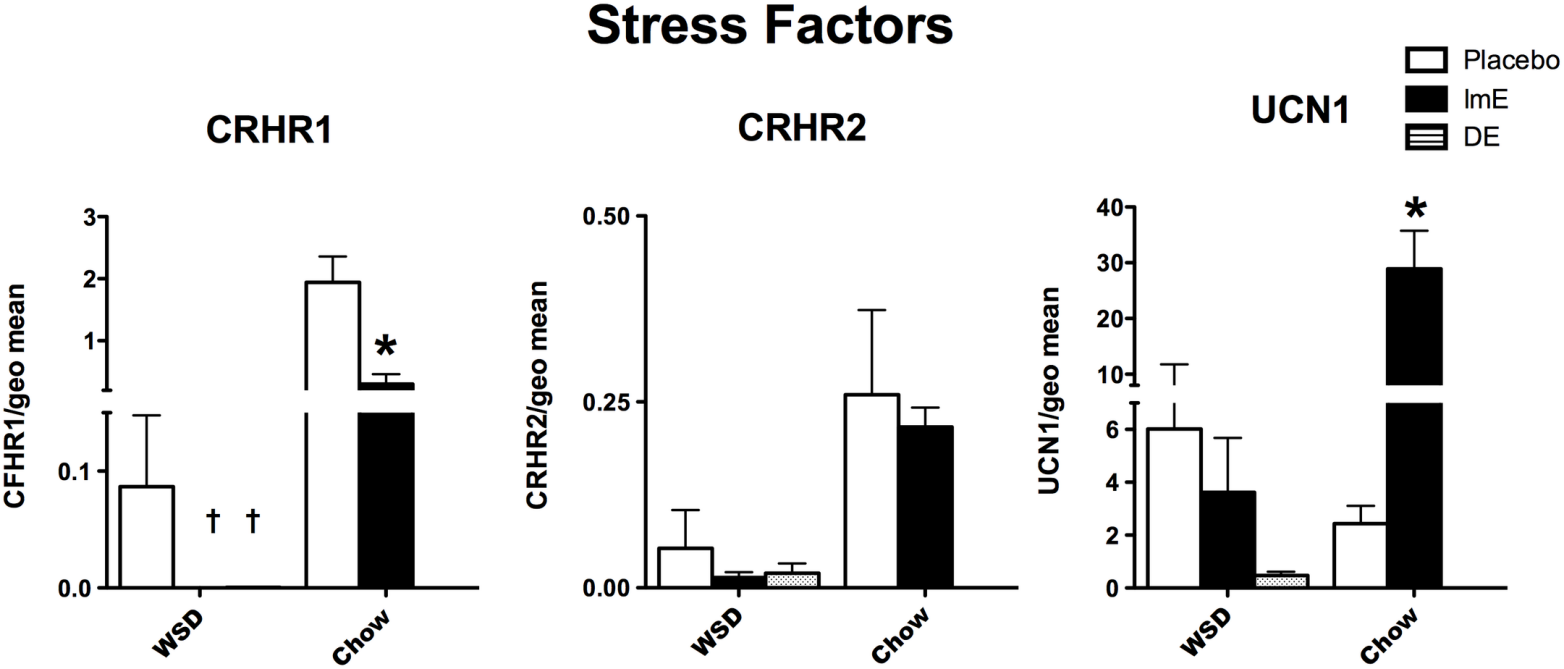
Histograms illustrating the expression of components of the CRH system in the midbrain. CRHR1 was significantly suppressed by all E treatments, regardless of diet. However, the overall expression of CRHR1 was 10-fold lower in WSD-fed groups. E–treatment did not regulate CRHR2 in either diet, and the overall transcript abundance was low in both diets. UCN1 was significantly increased by E-treatment in the chow-fed monkeys, but not in the WSD-fed monkeys. Thus, WSD prevented the UCN1 response to E. *significantly different from placebo (2-way ANOVA, Bonferroni p<0.001). † significantly different from placebo (1-way ANOVA, Newman-Keuls p<0.05).

The expression ratios of genes related to stress are illustrated in **Fig 3**. CRHR1 (anxiogenic) and UCN1 (stresscopin) showed an effect of diet (p = 0.0005; p = 0.013, respectively), an effect of E treatment (p = 0.0025; p = 0.007, respectively) and a significant interaction between diet and E treatment (p = 0.005; p = 0.002, respectively) as revealed by the 2-way ANOVA. In the chow group, there was a significant decrease in CRHR1 (Bonferroni p<0.001), and a significant increase in UCN1 (Bonferroni p<0.01) with E treatment. In the WSD-fed monkeys, CRHR1 mRNA was also significantly decreased with ImE and DE treatment compared to placebo treatment (1-way ANOVA F [2,12] = 4.772; p = 0.029; Newman Keuls posthoc p<0.05, both groups), whereas UCN1 was not different between the WSD-fed groups (p = 0.33).

There was no effect of E treatment on CRHR2 mRNA with either diet. However, earlier analysis of the chow-fed animals found that CRHR2 exhibited an elevated trend when the same data was normalized by GAPDH (*t* [5] = 2.008; p = 0.09). CRHR2 was a low abundance transcript in all monkeys.

### Synapse assembly

**Fig 4:**
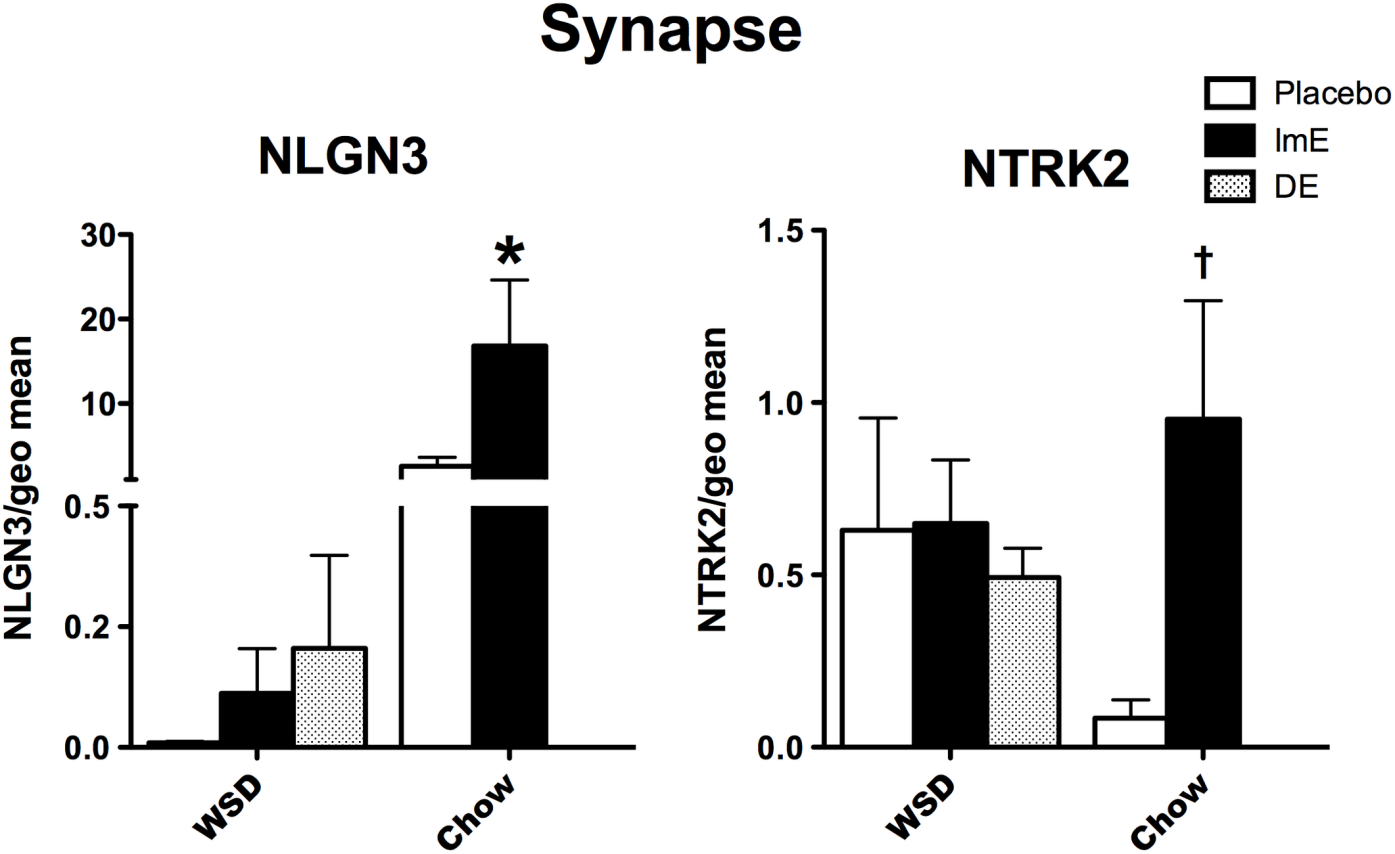
Histograms illustrating the expression of 2 genes that are involved in synapse assembly. NGLN3 and NTRK2 expression was increased with E treatment over placebo in chow-fed monkeys. However, ImE or DE had no effect on NLGN3 or NTRK2 mRNA expression in WSD-fed monkeys. Thus, WSD prevented gene response to E. * Bonferroni post hoc p<0.05. † t-test p<0.032.

The relative mRNA expression of 2 genes that are known to have pivotal roles in synapse assembly and maintenance is illustrated in **Fig 4.** NLGN3 expression showed an effect of diet (p = 0.006), an effect of E treatment (p = 0.031) and an interaction between diet and E treatment (p = 0.033). Transcript abundance was reduced in WSD-fed animals. NLGN3 was higher with E treatment in the chow-fed animals (Bonferroni p<0.05), but there was no response to E in the WSD-fed animals.

There was no effect of diet or E treatment on NTRK2, but there was interaction (p = 0.015) with 2-way ANOVA. However, NTRK2 mRNA showed an increase with E treatment over placebo in chow fed animals with a t-test (*t* [5] = 2.94; p = 0.032, respectively). There was no difference between treatment groups in the WSD-fed animals. Overall, NTRK2 transcript abundance was similar between WSD-fed and chow-fed monkeys.

### DNA repair

**Fig 5:**
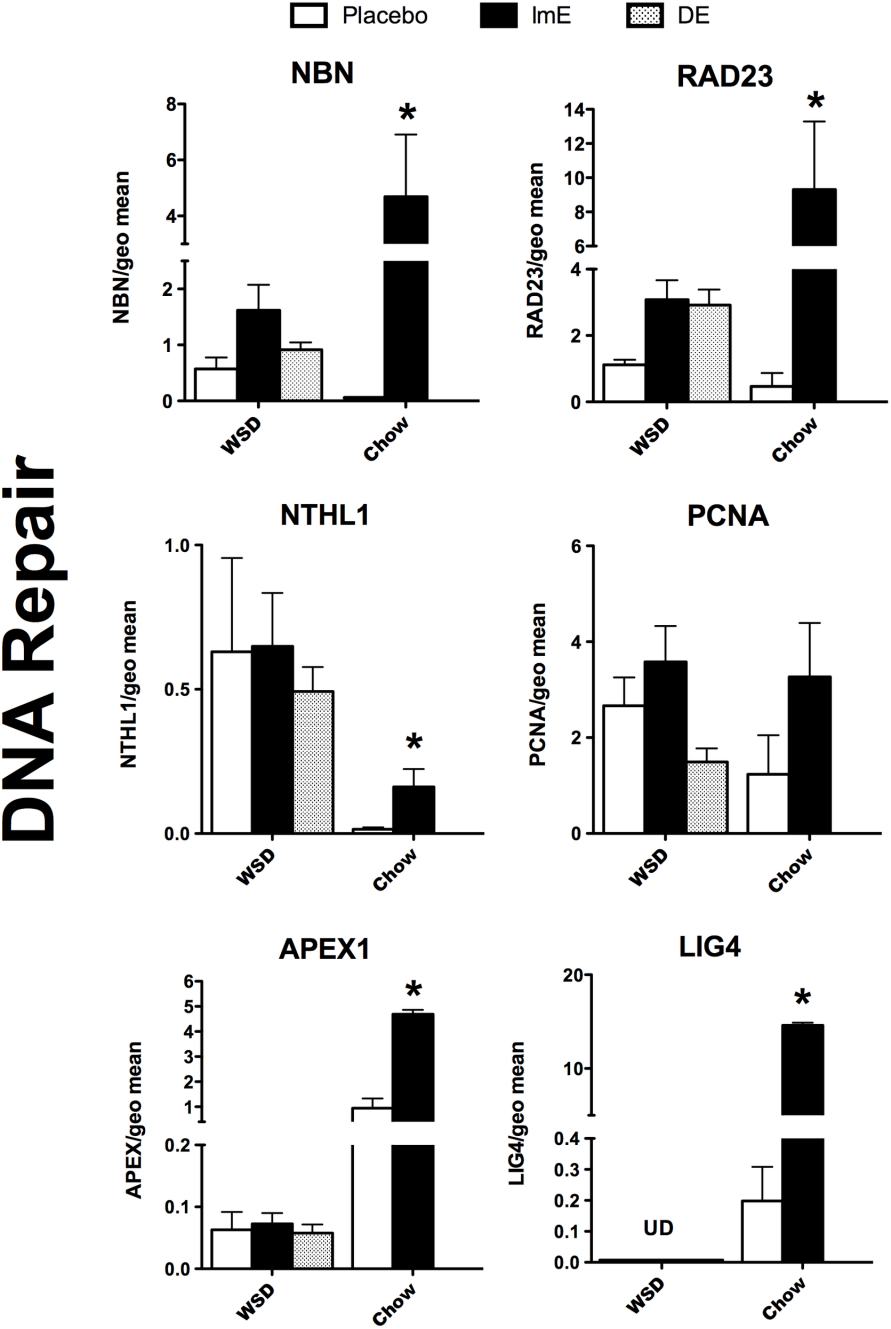
Histograms illustrating the expression of 6 genes encoding proteins that are involved in DNA repair. NBN, NTHL1, PCNA, APEX1, RAD23 and LIG4 mRNAs were increased by E-treatment in chow-fed monkeys. None of these genes showed a significant effect of ImE or DE in WSD-fed animals, although RAD23 exhibited an upward trend in a fashion similar to the chow fed monkeys. Four of the 6 genes exhibited lower transcript abundance with WSD than chow diet. * Bonferroni p<0.05.

Five of the 6 genes that promote DNA repair showed elevated expression with E treatment in the chow-fed monkeys (**Fig 5)**. With 2-way ANOVA, APEX1 expression showed an effect of diet (p<0.0001), an effect of E treatment (p<0.0001) and an interaction (p<0.0001). RAD23 expression exhibited an effect of E treatment (p<0.0046), no effect of diet, but an interaction between diet and steroid (p<0.05). NTHL1 mRNA exhibited an effect of diet (p<0.015), but no effect of E treatment or interaction between diet and E treatment. NBN mRNA showed an effect of E treatment (P<0.008), but no effect of diet and no interaction between E treatment and diet. PCNA mRNA was not different by 2-way ANOVA in diet, E treatment or interaction. LIG4 could not be analyzed with 2-way ANOVA. NBN, APEX and RAD 23 were higher in E-treated chow-fed animals compared to placebo (Bonferroni p<0.05). In WSD-fed animals, there was no difference between the groups in NBN, RAD23, NTHL1, PCNA or APEX1 with 1-way ANOVA. LIG4 was undetectable in the WSD-fed animals in stark contrast to the chow-fed animals. Overall expression of NBN, APEX1, RAD23 and LIG4 was 2 to10-fold lower in WSD-fed animals than in chow-fed animals.

### Neuroendangerment

**Fig 6:**
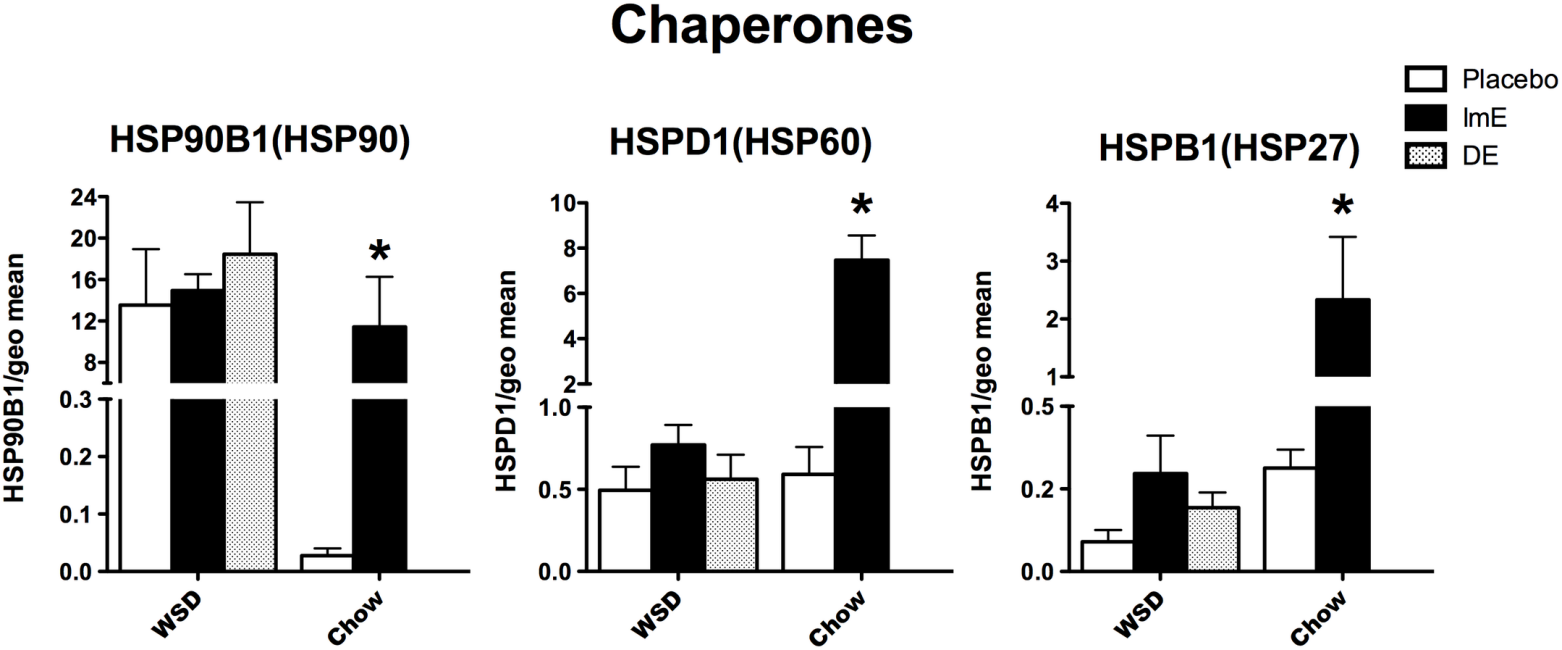
Histograms illustrating the expression of 3 genes that code for chaperone proteins involved in protein folding. HSP90, HSP60 and HSP27 mRNAs significantly increased with E- treatment in chow-fed monkeys. There was no regulation by ImE or DE in the WSD-fed monkeys, although HSR27 exhibited an upward trend. In addition, transcript abundance of HSP60 and HSP27 was reduced by WSD. * Bonferroni post hoc p<0.05.

Genes were examined in the categories of protein folding, protein degradation and transport. Sabotage of these functions is known to underlie different neurodegenerative diseases. Heat shock protein chaperones play a role in both DNA repair and steroid receptor functions. **Fig 6** illustrates relative mRNA expression of 3 chaperones of the heat shock protein family. HSP60 and HSP27 exhibited an effect of E treatment, diet and an interaction (p< 0.003 to 0.0001) with 2-way ANOVA. HSP90 exhibited an effect of diet (p<0.014), an effect of E treatment (p<0.05), but no interaction between the two. HSP90, HSP60 and HSP27 were increased by E treatment in the chow-fed monkeys with Bonferroni post hoc tests (p<0.05). There was no difference between treatment groups in WSD-fed monkeys with 1-way ANOVA. Overall expression of HSP60 and HSP27 was an order of magnitude lower in WSD-fed monkeys compared to chow-fed monkeys, but HSP90 mRNA was of similar magnitude in both diets.

**Fig 7:**
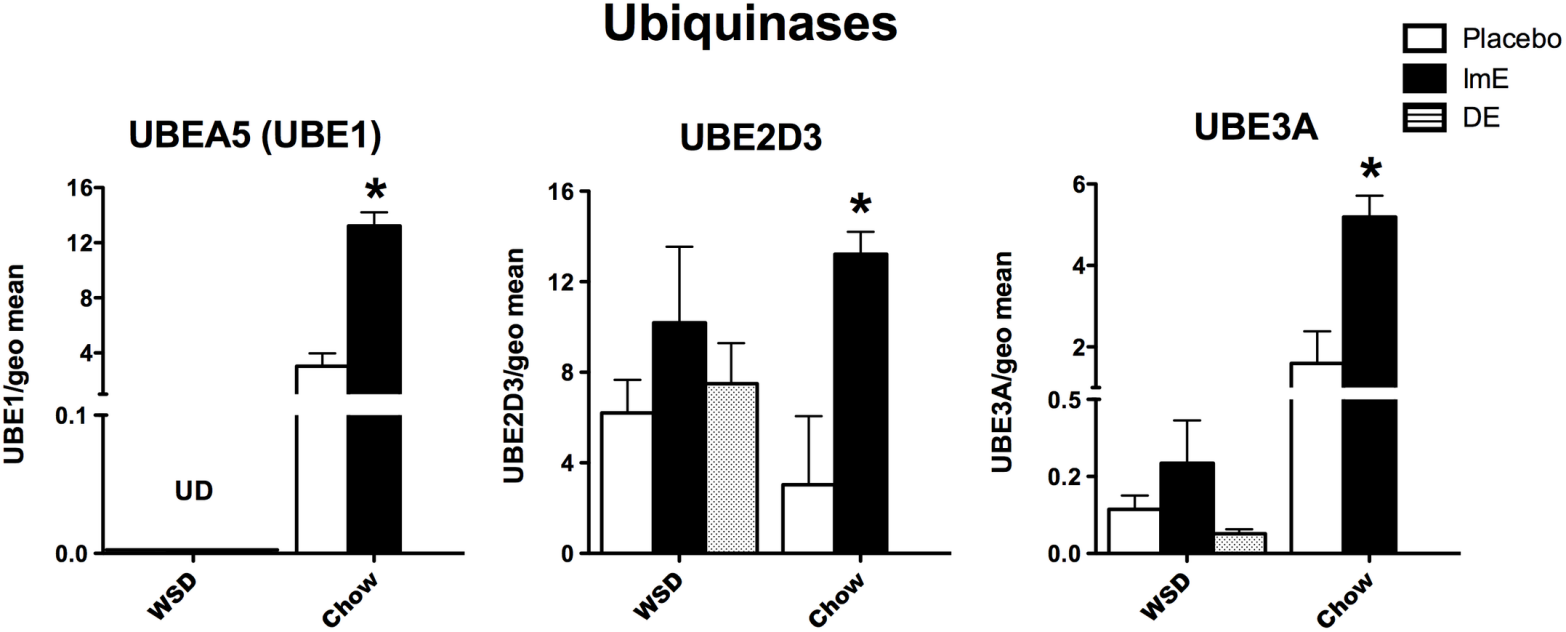
Histograms illustrating the expression of 3 genes that encode for ubiquinases, which are pivotal for protein degradation. Each ubiquinase significantly increased with E-treatment in the chow-fed animals. However, there was no difference between the groups in WSD-fed monkeys. In addition, transcript abundance was reduced by WSD for UBE1 and UBE3A. * Bonferroni p<0.001 or t-test p<0.01.

Ubiquinases, which govern protein degradation, have an important role in ridding the cell of faulty or damaged proteins, as well as regulatory molecules such as transcription factors. **Fig 7** illustrates mRNA expression of 3 genes that are representative of the 3 classes of ubiquinases. UBE1 (activating) and UBE3A (ligase) exhibited effects of diet, E treatment and an interaction (p<0.003 to 0.0001) with 2-way ANOVA. UBE2D3 (conjugating) showed a significant effect of E treatment (p<0.05), but no effect of diet and no interaction. UBE1, UBE2D3 and UBE3A mRNAs were significantly increased by E treatment over placebo treatment in the chow-fed monkeys (Bonferroni p<0.001). There was no difference between groups in the WSD-fed macaques (1 way ANOVA, p>0.05 all genes). UBE1 and UBE3A transcript abundance was an order of magnitude lower in the WSD-fed monkeys compared to the chow-fed monkeys.

**Fig 8:**
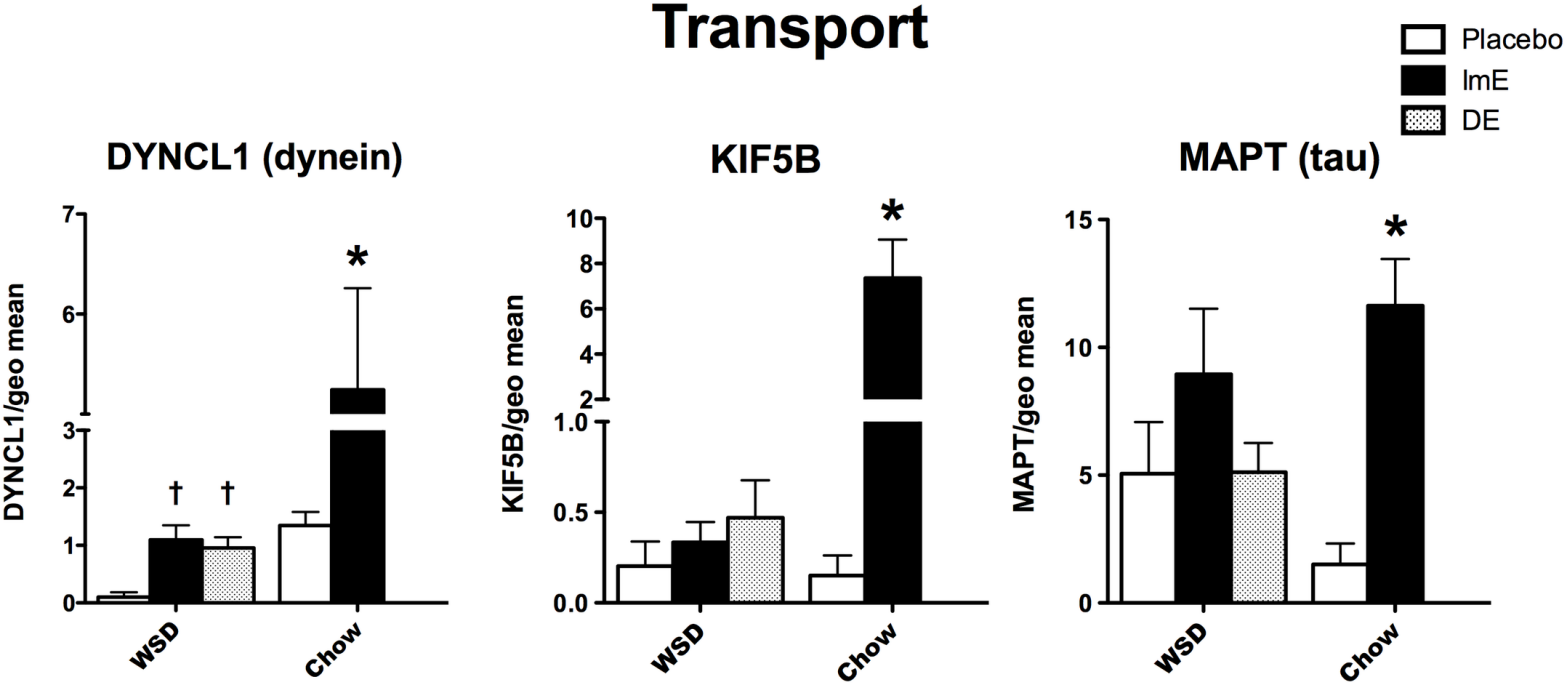
Histograms illustrating the expression of 3 genes that code for transport motor proteins. KIF5B, DYNCL1 and MAPT mRNAs were significantly increased by E-treatment in chow-fed monkeys. There was no significant difference between the groups for DYNCL1, KIF5B or MAPT in the WSD-fed monkeys. Both KIF5B and DYNCL1 transcript abundance was reduced in WSD- compared to chow-fed monkeys. * Bonferroni posthoc p<0.01. † Newman-Keuls p<0.05.

Cytoskeletal transport proteins play a role in Alzheimer’s and other neurodegenerative diseases. **Fig 8** illustrates the 3 genes examined that relate to cytoskeletal elements underpinning transport. Kinesin (KIF5B) and dynactin (DYNCL1) expression showed an effect of diet, E treatment and an interaction (p<0.007 to p<0.0001) with 2-way ANOVA. MAPT (tau) expression showed an effect of E treatment (p< 0.005), but no effect of diet and no interaction. All three mRNAs related to transport were increased with E treatment over placebo in the chow-fed monkeys (Bonferroni p<0.01), but only DYNCL1 increased in WSD-fed monkeys (1-way ANOVA p = 0.04). MAPT mRNA exhibited an elevated trend with ImE, but the variance precluded statistical significance. Overall, KIF5B mRNA was an order of magnitude lower and DYNCL1 mRNA was ~4-fold lower in WSD-fed monkeys than chow-fed monkeys.

### Neurodegenerative disease specific

**Fig 9:**
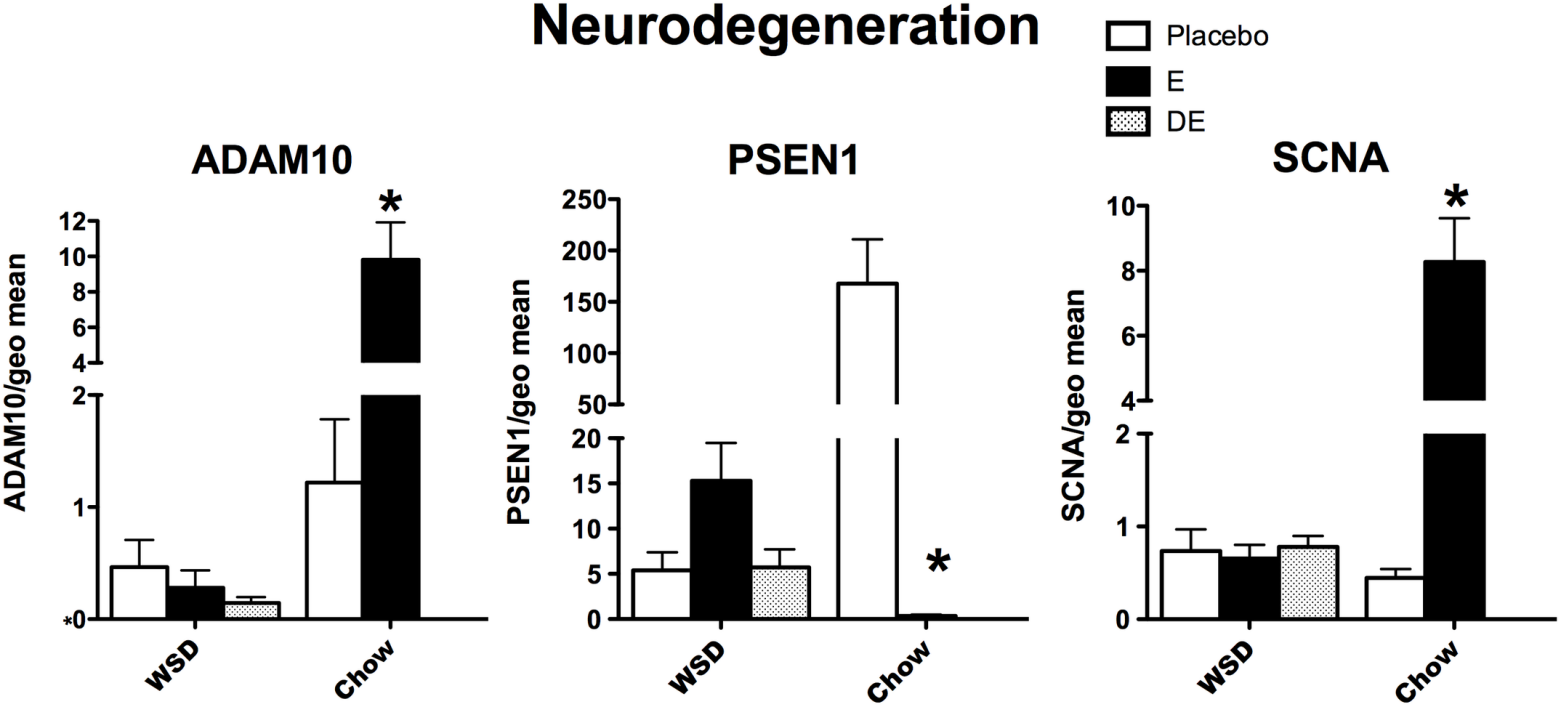
Histograms illustrating the expression of 3 genes encoding proteins known to be aberrant in different NDDs. ADAM10 and SCNA mRNAs significantly increased with E–treatment, whereas PSEN1 mRNA significantly decreased with E-treatment in the chow-fed monkeys. The effect of E observed in the chow-fed monkeys was lost in the WSD-fed monkeys. In addition, transcript abundance was decreased by an order of magnitude for ADAM10 and PSEN1 in the WSD- compared to chow-fed monkeys. * Bonferroni posthoc p<0.001.

It was of further interest to examine expression of representative genes that encode proteins known to be involved in neurodegenerative pathology. **Fig 9** illustrates 3 genes implicated in neurodegeneration. With 2-way ANOVA, ADAM10 (α-secretase), PSEN1 (γ-secretase) and SCNA (synuclein) expression showed an effect of diet, E treatment and an interaction (p<0.007 to 0.0001). ADAM10 and SCNA were higher, and PSEN1 was lower, with E treatment compared to placebo treatment in chow-fed monkeys Bonferroni p<0.001, all). Neither ADAM10, PSEN1 OR SCNA expression was different between the WSD-fed groups with 1-way ANOVA.

### Correlation with fat mass

Percent (%) fat mass of total mass was obtained from Dexascans of the individual animals at 6-month intervals. Correlation coefficients and linear regression analysis were obtained on the terminal % fat mass in (1) a gene that responded similarly to chow-fed animals and was stimulated by E (TPH2), and versus (2) a gene that was dissimilar to chow-fed animals and not stimulated by E (NTRK2). There was no correlation between the % fat mass and either TPH2 or NTRK2. There was no deviation of the linear regression line from zero.

### Further statistical results

In order for the text to be comprehensible, the full statistical results of all of the gene analyses, including F values, are shown in Table 3 in alphabetical order.

**Table 3:** ANOVA statistics for all genes.

Gene Name			2-way ANOVA on all groups (df = 1,12/effect)				1-way ANOVA on WSD (df = 2,12)	
	Diet		E treatment		Interaction			
	F	P	F	P	F	P	F	P
**5HT1A**	1.168	0.301	3.367	0.091	1.45	0.242	2.263	0.1466
**ADAM10**	7.835	0.0161	9.66	0.0091	11.42	0.0055	1.076	0.3718
**APEX**	166.7	<0.0001	78.17	<0.0001	77.34	<0.0001	0.2045	0.2045
**CRHR1**	22.68	0.0005	14.51	0.0025	11.75	0.005	4.865	0.0284
**CRHR2**	10.48	0.0071	0.4297	0.5245	0.0012	0.0727	0.8157	0.4654
**DYNCL1**	35.81	<0.0001	30.84	0.0001	10.28	0.0075	4.252	0.0402
**FEV**	51.15	<0.0001	26.65	0.0002	26.52	0.0002	0.8205	0.4635
**HSP90B1(90)**	8.183	0.0143	4.641	0.0522	2.823	0.1187	0.2552	0.7797
**HSPB1(27)**	7.438	0.0184	7.222	0.0198	4.789	0.0492	1.065	0.3751
**HSPD1(60)**	63.53	<0.0001	70.41	<0.0001	59.95	<0.0001	0.9797	0.4035
**KIF5B**	29.65	0.0001	33.08	<0.0001	30.7	0.0001	0.5291	0.6023
**MAPT**	0.6342	0.4382	1.932	0.1849	0.2145	0.6499	1.329	0.3042
**NBN**	1.917	0.1913	10.09	0.008	4.066	0.0667	2.079	0.1678
**NTHL1**	7.993	0.153	0.1812	0.6779	0.1081	0.748	0.4784	0.6321
**NTRK2**	0.2724	0.6112	3.624	0.0812	3.321	0.934	0.3509	0.711
**PCNA**	0.9765	0.3426	2.778	0.1215	0.3987	0.5396	1.236	0.325
**PSEN1**	10.57	0.0069	12.08	0.0046	15.29	0.0021	2.737	0.1049
**RAD23**	3.209	0.00985	12.04	0.0046	4.879	0.0474	2.482	0.1253
**SCNA**	48.67	<0.0001	55.4	<0.0001	57.4	<0.0001	0.2362	0.7935
**SERT**	20.79	0.0007	10.1	0.0079	10.35	0.0074	2.073	0.1686
**TPH2**	0.0986	0.7588	17.06	0.0014	1.676	0.2198	1.703	0.2234
**UBE1**	179.5	<0.0001	70.54	<0.0001	70.53	<0.0001	2.476	0.1258
**UBE2D3**	25.18	0.0003	9.899	0.0084	9.896	0.0084	0.5156	0.6098
**UBE3A**	45.24	<0.0001	15.82	0.0018	13.39	0.0033	1.599	0.2423
**UCN1**	8.505	0.0129	10.45	0.0072	15.04	0.0022	1.186	0.3387

## Discussion

### General

Since obesity has reached epidemic proportions, awareness has evolved that diet and excess adipose tissue increase inflammation through a variety of mechanisms [41]. Diet, obesity and inflammation can impact the functions of many physiological systems cumulating in the multiple symptoms of metabolic disease, which often includes depression [25,42]. This new knowledge led to our recognition that our monkey studies of the serotonin system were done with animals on normal monkey chow diet supplemented with fresh fruit and vegetables. Monkey chow is low in fat and refined sugar, but high in micronutrients. Together, all of the evidence raised important questions about the ability to translate our findings regarding sex steroid regulation of serotonin neurons in chow-fed monkeys to 40% of the women in the US that are obese [10,43]. With inconsistencies between human trials and animal responses to HT, the question of the interaction of obesity, diet and HT needed clarification.

We performed this long-term study in which old OvH rhesus macaques were maintained *ad libitum* for 2.5 years on a diet in which the same percent of calories were derived from fat and refined sugar as found in the typical western style diet (WSD). WSD-fed macaques exhibit weight gain and an increase in fat mass [44]. In addition, 3 treatment groups were established that mirrored the ‘window of opportunity’ hypothesis of HT. A previous study with old chow-fed monkeys and a similar design enabled comparisons old chow-fed monkeys and the old WSD-fed monkeys in this study. In both old chow-fed macaques and old WSD-fed macaques, RNA was extracted from a small microdissected piece of the midbrain containing the dorsal raphe nucleus.

The administration of E via Silastic capsule produced serum concentrations of E in the physiological range of the early follicular phase and approached those of women using a high dose vaginal ring (FemRing^TR^). Of note, the chow-diet in the comparison experiment contained soybean meal, which contains phytoestrogens. Macaques can metabolize phytoestrogens to S-equol probably via intestinal flora [45,46], and S-equol binds to ERβ [46], the receptor in serotonin neurons [47]. Phytoestrogens can also inhibit aromatase activity. Nonetheless, in the chow-fed monkeys, the exogenous administration of E would bypass aromatase and E would fully activate ERα and ERβ, rendering the activity of S-equol to little functional consequence. In the placebo-treated group, the possibility that there was some stimulation of ERβ cannot be ruled out, but it clearly did not approach the effect of exogenous E treatment. There was no soybean meal in WSD, although miniscule amounts of isoflavones may be present in minor wheat and corn components.

In addition to the independent variable of diet, there were 4 other variables that differed between this study and the previous study with old chow-fed macaques [1] length of treatment (chow = 48 months versus WSD = 30 months or 6 months), [2] surgery (chow = Ovx versus WSD = OvH), and [3] housing (chow = paired housing versus WSD = pen housing) and [4] minor difference in interval to E treatment (chow = 2 months versus WSD = immediately). We previously found little or no difference in the patterns of gene expression between laser captured serotonin neurons from adult Ovx monkeys with 1-month of E treatment and old Ovx monkeys with 48-months of E treatment [33]. Therefore, we suggest that these 4 variables are minor compared to the effect of diet, and that comparing the results from the different diets is informative.

In order to determine the significance of the interaction between diet and E treatment, 2-way ANOVA was required. However, 2-way ANOVA required an equal number of groups and it would not work on the 5 groups from which data were obtained. This necessitated executing the 2-way ANOVA on only the placebo and ImE groups across the 2 diets. Further analysis was required in which a 1-way ANOVA was applied across the 3 treatment groups of the WSD-fed animals. On two occasions when the Bonferroni test did not show a significant difference between placebo- and E-treated chow-fed animals, but we previously published a difference [33], then the chow-fed groups were examined with a t-test and the results were included. Nonetheless, the 2-way ANOVA assigned significance to the difference in transcript abundance between the diets and assigned significance to the interaction between diet and treatment. The definitive nature of these results made the 2-way analysis useful.

### Serotonin system

In the old chow-fed monkeys, E increased FEV, TPH2 and SERT mRNAs. In WSD-fed monkeys, E did not increase FEV expression. In addition, there was no statistical difference in TPH2 or SERT across the WSD groups although the upward trend with E treatment was similar to chow-fed animals. The ability of the WSD-fed monkeys to mount even a blunted response to E for TPH2 differs from our observation in marmosets, which had absolutely no response to E on a high fat diet [48]. Of note, indoleamine 2,3-dioxygenase (IDO) mRNA trended higher in the WSD-fed placebo group compared to the ImE or DE groups (ratio equaled 144.0±135.5, 0.28±17.28 and 2.33±0.82, respectively), but the differences were not significant due to extreme variance. IDO was not determined in the chow-fed animals. Nonetheless, the data concur with the idea that depression in metabolic disease may involve cytokine actions that switch the metabolic pathway of tryptophan from THP2 to IDO, and the formation of neurotoxic kynurenines [49]. This trend is mentioned because a type 1 error due to the small ‘n’ would negate what may be an important observation.

Since depression has been linked to metabolic disease [50–52], we expected decreased serotonin production in obese animals on WSD. However, emotional eating and food preference tests suggest that serotonin release may be part of a food reward circuit [53–55] in which carbohydrates release serotonin and improve mood [53,54,56]. Numerous studies have shown that E is anti-inflammatory and blocks the effects of cytokines in different tissues [42,57–61]. Overall, high fat diet-induced cytokines may hinder serotonin production whereas E and carbohydrates may promote increased serotonin production. Furthermore, the balance of these opposing factors may shift as a person becomes heavier with time or with the age-related decline in metabolic activity.

E did not affect 5HT1A mRNA expression in the chow-fed monkeys and 5HT1A was highly variable in the WSD-fed monkeys. This differs from the reduced expression of 5HT1A mRNA and protein observed with in situ hybridization (ISH) and binding assays in adult chow-fed females ± 1 month of ERT [62,63]. The ISH and binding assays used coronal slices of the midbrain raphe and only autoreceptors were measured in serotonin neurons. However, the block preparations from the old monkeys contain both autoreceptors and post-synaptic 5HT1A receptors, which could be regulated differently. Any regulation in the autoreceptors could have been obliterated by the expression of the post-synaptic receptors.

Therefore, WSD blunted the ability of E to increase FEV, which determines the serotonin phenotype [64]. However, the ability of E to slightly increase TPH2 and SERT expression, albeit in a variable fashion [52], in WSD-fed monkeys suggests that E may retain some beneficial effects in the serotonin system of obese individuals, within the time frame of these experiments.

### CRH system

CRHR1 is the anxiolytic receptor that binds CRH and decreases serotonin release. CRHR1 mRNA decreased in both chow-fed and WSD-fed old monkeys with each E-regimen. Of note, the decrease in CRHR1 in the WSD-fed animals was similar between ImE and DE. If this data reflects serotonin neurons, then it is consistent with the notion that the serotonin system retains some responsiveness to E after a long interval. Thus, E removed a ‘brake’ on serotonin neurons, which could act to reduce anxiety. In contrast, there was no significant effect of any E-regimen on CRHR2 in chow-fed or WSD-fed old monkeys, which differed from the increase observed in laser-captured serotonin neurons [65]. Also, CRHR2 was a very low abundance transcript in these block preparations compared to the laser-captured preparations. UCN1 (stresscopin) was significantly increased with E treatment in chow-fed old macaques, but not in WSD-fed old macaques. This could contribute to lowered stress resilience in obese individuals.

### Synapses

Much of adult plasticity relies on extrusion and retraction of dendritic spines. Furthermore, the development of new synapses on dendritic spines has been linked to the expression of glutamate receptors [66,67]. E treatment increased the expression of 2 genes that code for selected synapse assembly proteins in chow-fed old monkeys. However, E-stimulation was lost in WSD-fed old monkeys and the overall expression of NLGN2 was greatly reduced with the obesogenic diet. If this is a representative response, it may not bode well for other synaptic proteins or glutamate receptors. Although E did not regulate NTRK2 (TrkB) with the obesogenic diet, the overall expression was comparable in WSD-fed and chow-fed old monkeys. Interestingly, functional characterization of human NTRK2 mutations were identified in subjects with severe early-onset obesity [68] and NTRK2 was involved in appetitive behavior [69]. In addition, NTRK2 knockout mice developed obesity and hyperphagia [70]. Also, an association study found evidence of NTRK2 linked to childhood-onset mood disorders [71]. The loss of E–induced NTRK2 mRNA suggests that its transcription may be highly sensitive to obesogenic diets.

### DNA repair

The absence of ovarian steroids led to serotonin neuron degeneration in young adult Ovx macaques. This was an important observation since there was no overt physical trauma to the brain [31]. DNA repair minimally requires lesion recognition, single strand excision, lesion removal, gap-filling synthesis, and finally ligation [72]. In the old chow-fed Ovx monkeys, 5 of 6 genes needed for DNA repair were significantly higher with E treatment. This was similar to the response observed in laser-capture preparations from younger, adult chow-fed Ovx monkeys [32]. In the old WSD-fed monkeys, none of the genes responded to E. NBN and RAD23 were elevated, but the variance precluded a statistical significance. NTHL1 and APEX showed no response to ImE or DE compared to placebo controls. PCNA showed an elevated trend with E-treatment in the chow-fed animals, but the opposite was observed in the WSD-fed animals. That is, ImE had no effect and DE had a suppressive effect. Other than an odd effect of diet, the responses of PCNA are hard to interpret. LIG4 was significantly increased by E treatment in the chow-fed animals, but it was undetectable in the WSD-fed animals. Overall expression of the DNA repair genes was 2-fold to an order of magnitude lower for 4 genes in WSD-fed groups compared to chow-fed groups. This collection of 6 genes is barely representative of all of the proteins involved in DNA repair [73–75]. Nonetheless, the obesogenic diet reduced transcript abundance for 4 genes, and eliminated up-regulation by E for 5 genes of the 6 genes examined. Together the data suggest that DNA repair may be hindered by an obesogenic diet.

### Underlying mechanisms of neurodegenerative diseases (NDDs)

The common NDDs observed in aging human populations have *not* been observed in female macaques maintained on normal primate chow. Nonetheless, a number of NDDs involve translation of normal genes whose proteins normally subserve important cellular functions, but with disease, the proteins are mis-folded, mis-processed or subverted from normal transport [76].

HSPs are chaperones that are widely known for their role in sex steroid receptor functions, but they also play pivotal roles in DNA repair and neuroprotection [77–82]. E significantly increased HSPs 90, 60, and 27 in the old chow-fed monkeys. However, the responses to ImE and DE were blunted in the old WSD-fed monkeys. HSP27 showed an elevated trend with ImE, but there was no effect on HSP90 or HSP60. In addition, the overall expression of HSP60 and HSP27 was 6-fold to an order of magnitude lower in the WSD-fed females. Loss of chaperone expression or function has destructive downstream consequences for protein folding and degradation, for sex steroid receptor function and for DNA repair.

Similar results were observed with representative ubiquinases that support activation, conjugation and ligation functions. Protein ubiquitylation has emerged as an important regulatory mechanism that impacts almost every aspect of the DNA damage response [72,83–85]. Long-term E treatment significantly increased each ubiquinase in the old chow-fed monkeys, but neither ImE nor DE treatments had significant effects in the WSD-fed monkeys. Blunted trends toward higher mRNA expression were observed, but overall expression of UBE1 and UBE3A was 1 to 2 orders of magnitude lower in WSD-fed monkeys compared to chow-fed monkeys. Lack of proper ubiquitylation leads to accumulation of degraded or misfolded proteins. This is particularly evident in plaques and tangles of Alzheimer’s disease and aggregation of α-synuclein with Parkinson’s disease [86,87].

Axonal transport defects also play a role in neurodegeneration [88–91]. KIF5B, which codes for kinesin (anterograde), DYNCL1, which codes for dynein (retrograde) and MAPT (tau, a microtubule associated protein) were significantly increased with E treatment in old chow-fed monkeys, but not in WSD-fed monkeys. In addition, the overall expression of KIF5B and DYNCL1 was decreased from 8 to 10 fold. The dysregulation of these transport genes could have important ramifications for Alzheimer’s disease in our aging population.

### Disease specific

A number of proteins have been implicated in certain neuropathies, but they also play important roles in normal functioning. As seen in the chow-fed animals, E-induction of ADAM10 mRNA (α-secretase) would improve correct processing of amyloid precursor protein, and E-suppression of PSEN1 mRNA, (component of γ-secretase that produces Aβ), would prevent plaque formation. In contrast, there was no response to E in the WSD-fed females. Epidemiological observations indicated that HT may delay the onset of Alzheimer’s [92], but the variability in this observation from trial to trial could be due to varying numbers of obese women in each trial. Synuclein (SCNA) is important for synaptic function and the E-induced increase in expression in the chow-fed monkeys would be a sign of improved synaptic function. SCNA had no response to ImE or DE in the WSD-fed monkeys, suggesting that a beneficial effect of HT was blunted with the obesogenic diet.

### ImE versus DE and transcript abundance

Two mRNA’s (CRHR1 and DYNCL1) that exhibited a significant effect of E treatment in the WSD-fed groups showed the same response to ImE and DE although CRHR1 mRNA decreased and DYNCL1 mRNA increased. This raises the question of whether rhesus macaques may lack a ‘window of opportunity’ with regard to the serotonin response to E replacement. Overall, only 2 genes in the WSD-fed animals exhibited transcription that was similar to the chow-fed animals in a statistically significant manner.

The descriptions of transcript abundance were based on the ability of the 2-way ANOVA to detect a difference in expression due to diet. Although every effort was made to execute the RT-PCR reactions in a similar manner, there could have been assay variables that contributed to differences in transcript abundance such as newer availability of Universal Taqman PCR mix or Preamp Mix, etc. rather than the use of individual reagents.

The lower transcript abundance in many of the genes examined in the WSD-fed animals also raised the possibility that increased lipid content of the brain interfered with the RNA extraction or in reverse transcription or that something caused a difference in assay sensitivity. However, one would expect the ratio of gene/housekeeping gene would remain the same regardless of the total RNA extracted. Moreover, some genes showed similar or elevated transcript abundance in WSD-fed compared to chow-fed monkeys. This argues for individual gene responses to WSD rather than an unknown blanket variable. Nonetheless, it is worrisome for human health that many of the ratios declined so greatly with WSD.

These caveats suggest that focus on the pattern of expression rather than the absolute values leads to a more conservative or cautious interpretation, but they do not alter our conclusion that WSD interferes with the ability of E to regulation gene expression in a normal fashion. Experiments are needed in which animals can be maintained on chow and WSD at the same time and processed for gene expression in the same assay. Examination of transcript abundance in human tissue is also warranted.

## Conclusions

Throughout this report, we have referred to an E-induced increase in gene expression. While this is an accurate description of the histograms, it is physiologically more plausible that gene expression decreased with OvH or Ovx and that E-treatment *maintained* optimum expression. An obesogenic diet for 2.5 years in old surgically menopausal macaques blunted or increased variability in E-induced gene expression in the dorsal raphe. In addition, the overall expression of many of the genes was lower in the WSD-fed compared to the chow-fed females. The loss of E-regulation of pivotal amyloid protein processing enzymes, as well as lack of proper synapse assembly, DNA repair, protein degradation, protein folding and transport functions suggests that HT may not be as beneficial to neural function and viability in obese women compared to normal weight women; and that WSD with obesity could foster development of Alzheimer’s disease. However, the serotonin-related genes maintained some responsiveness to E treatment with the obesogenic diet.
